# The future of *Viscum album* L. in Europe will be shaped by temperature and host availability

**DOI:** 10.1038/s41598-022-21532-6

**Published:** 2022-10-12

**Authors:** Łukasz Walas, Wojciech Kędziora, Marek Ksepko, Mariola Rabska, Dominik Tomaszewski, Peter A. Thomas, Roman Wójcik, Grzegorz Iszkuło

**Affiliations:** 1 Institute of Dendrology, Polish Academy of Sciences, Parkowa 5, 62-035 Kórnik, Poland; 2 Institute of Forest Sciences, Warsaw University of Life Sciences – SGGW, ul. Nowoursynowska 159, 02-776 Warszawa, Poland; 3 Bureau for Forest Management and Geodesy, ul. Lipowa 51, 15-424 Białystok, Poland; 4 School of Life Sciences, Keele University, Staffordshire, UK; 5 Institute of Biological Sciences, University of Zielona Góra, Prof. Z. Szafrana 1, 65-516 Zielona Góra, Poland

**Keywords:** Biogeography, Forest ecology

## Abstract

*Viscum album* L. is a plant of great importance due to its influence on the host trees and, by extension, entire ecosystems. The species is also significant to humans—on the one hand, because of its use in medicine, and on the other, because of the growing threat it poses to the stability of conifer stands. Therefore, it is important to recognize the future range of three mistletoe subspecies (*Viscum album* subsp. *album*, *V. album* subsp. *austriacum*, and *V. album* subsp. *abietis*). Modelling of the potential range of these subspecies was performed using MAXENT software. Locations were collected from literature and databases. A total number of 3335 stands were used. Bioclimatic data for the current conditions and three future scenarios (SSP 1.26, SSP 3.70, SSP 5.85) were downloaded from the CHELSA database. The results confirmed that the temperature is the key variable on the potential range of the analysed subspecies. *V. album* subsp. *abietis* is withdrawing from its range according to all scenarios. In the case of *V. album* subsp. *austriacum*, a slight range shift is visible. Only the *V. album* subsp. *album* will expand non-directionally. The reason is most likely a very large number of host species and greater genetic variability compared to the subspecies found on conifers.

## Introduction

The distribution of plants and the composition of forest ecosystems are strongly influenced by climatic conditions, which are key factors shaping the ranges of individual species. Future climate change poses a threat to many taxa, while for others it may represent an opportunity to expand their range^1,2^. Thus, rapid environmental changes in the near future could have a significant impact on the structure of the forests of Europe, assessing probable range shifts crucial to planning future forest management^3,4^. Currently, a frequently used method to determine changes in the potential ranges of various species is Species Distribution Modelling (SDM), which has become an important analytical tool in conservation biology, land management and spatial ecology^5,6^. One of the widely used algorithm is the maximum entropy algorithm, implemented in MAXENT. This software uses current locations of populations and a set of selected environmental variables^7–9^, allowing estimation of future distribution of rare^10^ or widespread taxa, such as tree species that form the core of European forests^3,4^. The MAXENT results also provide a better understanding of which factors most influence the occurrence of analysed species.

An example of a species that has a great influence on forest communities is *Viscum album* L. (mistletoe). It is a hemi-parasite, which has a significant impact on single trees, and, by extension, on forest ecosystems. Mistletoe may adversely affect the host tree, causing weakness, a decline in growth rate and even tree dieback^11–13^. The effect on trees in dry periods is particularly negative, because the mistletoe, unlike the host, does not close the stomata, increasing the water deficit of the host plant^14–17^. In recent years, *V. album* has become a more significant threat, especially to coniferous forests in Europe due to its significant and rapid expansion^18–21^. The greater presence of birds attracted by *Viscum* fruit, the increased amount of light reaching the forest floor due to host dieback, and the fall of *Viscum* leaves rich in minerals, cause the transformation of forest communities^22–24^. It is also a culturally significant plant that has been collected for Christmas decorations and medicinal use for centuries^25^. *V. album* has several pharmacological properties; the most important active substances are the viscotoxins and mistletoe lectins^26,27^.

Mistletoe prefers a mild, oceanic climate with summer temperatures above 15  °C and winter temperatures not less than − 7 °C^25,28^. During warm periods of the Holocene, this species was more widespread in northern Europe than is today, as evidenced by pollen data from Scandinavia^29^. The observed increase in mistletoe frequency in Central Europe during the last few years is probably related to the ongoing climate changes. As these changes are primarily associated with a higher average annual temperature, it may significantly increase the suitability for the mistletoe in the northeastern part of its range^28^. It is, therefore, possible for this species to expand north and east into Scandinavia and Russia while disappearing from South Europe, which will probably become too warm. There may also be an upward migration of mistletoe in mountainous regions, as it was observed for *Viscum album* subsp. *austriacum*—in the Swiss Alps its altitudinal range has already changed by about 200 m over the last century^30^ and by 200–300 m in southern Spain^31^. However, because *Viscum* is a hemi-parasite, its range is also influenced by the presence of a suitable host. Remarkably, the different subspecies differ as to the tree species that can fulfil this role^32^.

*Viscum album* is classified into five subspecies, from which four occur in Europe and one, *Viscum album* subsp. *meridianum* (Danser) D.G.Long, is native to E Himalaya to China and N Indo-China^25,33,34^. In Europe, the following subspecies are recognised: *Viscum album* L. subsp. *album, V. album* subsp. *austriacum* (Wiesb.) Vollm., *V. album* subsp. *abietis* (Wiesb.) Abrom., and endemic for Isle of Crete *V. album* subsp. *creticum* N. Böhling, Greuter, Raus, B.Snogerup, Snogerup & Zuber^25^. These taxa are very similar morphologically, but differ concerning the host and are distinguishable by molecular methods^35^. *Viscum album* subsp. *album* has the widest distribution and has been recorded on 384 deciduous tree species^32^. The other subspecies all occur on coniferous trees, i.e. *V. album* subsp. *austriacum* mainly on *Pinus sylvestris* L., *V. album* subsp. *abietis* mainly on *Abies alba* L. and *V. album* subsp. *creticum* on *Pinus brutia* Ten.^25,33^. There are also biochemical differences between the host races^36^. The subspecies are unlikely to cross with each other, and a hybrid has been described only between *V. album* subsp. *abietis* and *V. album* subsp. *album*^25,35^. Despite some differences among these taxa, the taxonomic status of the subspecies is still not clear and their identification relies almost exclusively on the identification of their hosts^37^.

Recently, mistletoe has become a plant of relatively high interest, and a large number of papers have been published on this species covering morphology, chemistry, genetics, and occurrence^25,32,35,36,38–42^. To date, however, no work has been published on modelling the potential future European range of this species, which would allow us to better understand the currently observed dynamics of mistletoe spread and to plan strategies related to the management of forests threatened by this hemi-parasite^28,43^. The aim of the presented analyses was to determine the potential current and future range of three mistletoe subspecies from Europe (*Viscum album* subsp. *album*, *V. album* subsp. *austriacum*, and *V. album* subsp. *abietis*). Fourth European subspecies, *V. album* subsp. *creticum*, was omitted due to its very restricted range.

## Materials and methods

### Data collection

Data on *Viscum album* occurrences were collected from databases (GBIF.org^44,45^: https://doi.org/10.15468/dl.zw6f5q; https://doi.org/10.15468/dl.6wmc9d; FloraWeb^46^; Pladias^47^); and literature^48–52^. In total, 130,430 sites were identified for the whole of Europe. Because a large proportion of these sites were very close to each other, or even repeated, to avoid bias only one stand for a particular subspecies was used for every 0.1° for *V. album* subsp. *austriacum* and *V. album* subsp. *abietis*, whereas for the most widespread *V. album* subsp. *album* one stand for every 0.5° was left. Thus, 3335 points remained: 1541 for *V. album* subsp. *album*, 1003 for *V. album* subsp. *austriacum* and 791 for *V. album* subsp. *abietis*.

### Niche modelling

The potential range of each subspecies was estimated using the maximum entropy algorithm implemented in MaxEnt 3.4^7,8^. The 19 bioclimatic variables for both current climatic conditions and future scenarios were downloaded from the CHELSA database^53^ with a resolution of 30 arc-sec. Climate projections obtained from the Coupled Model Intercomparison Project Phase 6 (CMIP6) were used to assess possible future changes in the subspecies' range. Three scenarios for the period 2041–2070 (SSP 1.26, SSP 3.70, SSP 5.85) were tested using the MPI-ESM1-2 model^55^. The SSP scenarios (Shared Socioeconomic Pathways) are based on socio-economic developments models. SSP 1.26 assumes a "green road" and a reduction in CO_2_ emissions to zero in about 2075; SSP 3.70 envisions economic competition between countries, resulting in a doubling of emissions before the end of the twenty-first century. The most pessimistic SSP 5.85 scenario assumes a tripling of CO_2_ emissions by 2075. Correlations between variables were calculated using the *raster* package in the R environment^56,57^. Finally, 11 uncorrelated variables were used as input. Occurrences of potential hosts were not used as variables in the models, but their potential range^58^ was compared with the results obtained for mistletoe.

Analyses were conducted as a bootstrap with 100 replications, 10,000 iterations, and a 10^–5^ convergence threshold. For each replication, 20% of the data were used as the test points with the ‘random seed’ option. Each model was evaluated using the receiver operating characteristic curve (ROC) and the area under the curve (AUC)^59,60^. The results of the modelling were visualised in QGIS 3.16.4 ‘Hannover’^61^.

## Results

**Figure 1:**
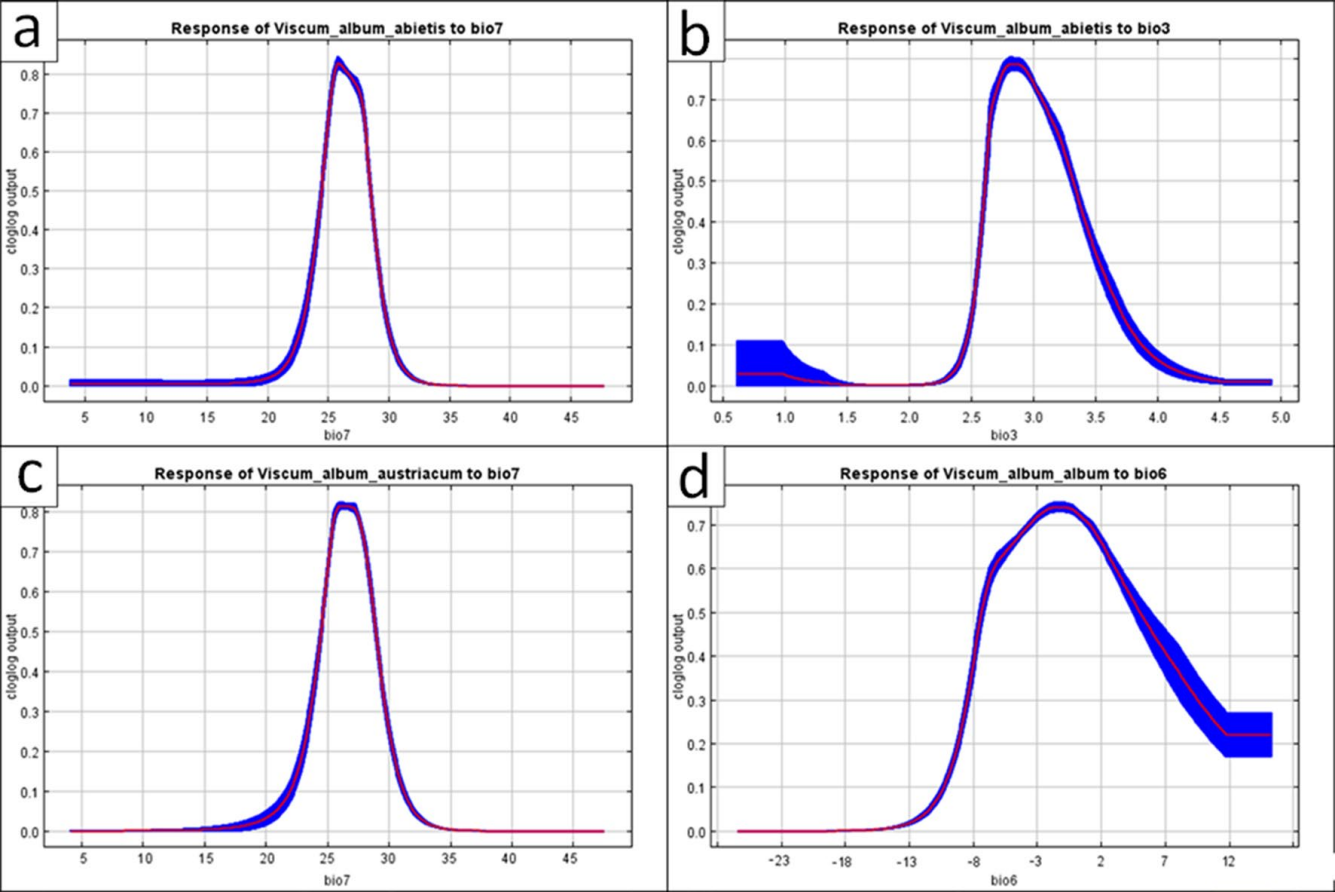
Response of *Viscum album* subspecies to the most important environmental variables (above 30% of contribution); (**a**) response of *V. album* subsp. *abietis* to temperature annual range, (**b**) response of *V. album* subsp. *abietis* to isothermality, (**c**) response of *V. album* subsp. *austriacum* to temperature annual range, (**d**) response of *V. album album* to the minimum temperature of the coldest month.

MaxEnt models based on the collected set of locations gave good predictions of the distribution of the three subspecies of *V. album*. All models tested achieved AUC greater than 0.8, which indicates a good fit to the data set. For all subspecies, temperature factors shape the potential range more strongly than precipitation (Table 1, Fig. 1a–d). The most important variables vary between subspecies, but one factor, bio7 (Temperature Annual Range) was significant in all models, especially for *V. album* subsp. *austriacum* (Fig. 1c), for which it was the most important, along with bio3 (Isothermality). For *V. album* subsp. *album*, the most important variables were bio6 (Minimum Temperature of Coldest Month, Fig. 1d), bio7, and bio17 (Precipitation of Driest Quarter). For *V. album* subsp. *abietis* the most significant factors were bio3 (Isothermality) and bio7 (Fig. 1a,b); also important were bio6 (Minimum Temperature of Coldest Month) and bio18 (Precipitation of Warmest Quarter).

**Table 1:** Contribution (%) of the most important bioclimatic variables in the tested climate models.

Taxa	*V. album* subsp. *abietis*				*V. album* subsp. *austriacum*				*V. album* subsp. *album*			
Scenario	Current	SSP 1.26	SSP 3.70	SSP 5.85	Current	SSP 1.26	SSP 3.70	SSP 5.85	Current	SSP 1.26	SSP 3.70	SSP 5.85
AUC	0.942	0.941	0.941	0.941	0.922	0.921	0.92	0.923	0.814	0.817	0.814	0.815
bio2	5.9	5.0	5.9	5.0	3.1	3.1	3.2	3.1	1.1	1.0	0.8	0.9
bio3	**32.3**	**34.3**	**33.3**	**34.6**	**27.4**	**27.3**	**27**	**26.7**	2.6	4.2	2.7	5.1
bio5	3.2	3.0	2.4	3.4	6.8	6.3	6.4	5.6	5.4	4.9	5.2	5.1
bio6	**12.6**	**11.0**	**12.7**	**10.8**	4.1	4.7	3.6	4.0	**48.5**	**44.6**	**48.7**	**44.0**
bio7	**32.7**	**32.2**	**32.5**	**30.5**	**45.5**	**45.8**	**46.0**	**46.3**	**22.9**	**26.7**	**24.0**	**26.9**
bio8	1.0	0.8	0.5	0.7	5.2	5.9	6.1	7.0	0.3	0.2	0.3	0.4
bio9	0.2	0.3	0.2	0.2	0.2	0.5	0.2	0.2	0.1	0.1	0.1	0.1
bio15	0.7	0.6	0.6	0.7	0.3	0.4	0.6	0.3	0.5	0.7	0.5	0.6
bio17	0.2	0.2	0.1	0.3	4.8	3.8	4.5	4.7	**15.6**	**14.9**	**15.1**	**14.2**
bio18	**11.1**	**11.9**	**11.5**	**13.3**	0.4	0.6	0.6	0.6	2.4	1.9	1.9	1.8
bio19	0.1	0.7	0.4	0.5	2.2	1.6	1.9	1.5	0.6	0.8	0.6	0.9

*AUC* area under the curve, variables presented in the table are as follows: bio2 (Mean Diurnal Range of Temperature), bio3 (Isothermality), bio5 (Maximum Temperature of Warmest Month), bio6 (Minimum Temperature of Coldest Month), bio7 (Temperature Annual Range), bio8 (Mean Temperature of Wettest Quarter), bio9 (Mean Temperature of Driest Quarter), bio15 (Precipitation Seasonality), bio17 (Precipitation of Driest Quarter), bio18 (Precipitation of Warmest Quarter), and bio19 (Precipitation of Coldest Quarter). The most important variables are bolded.

**Figure 2:**
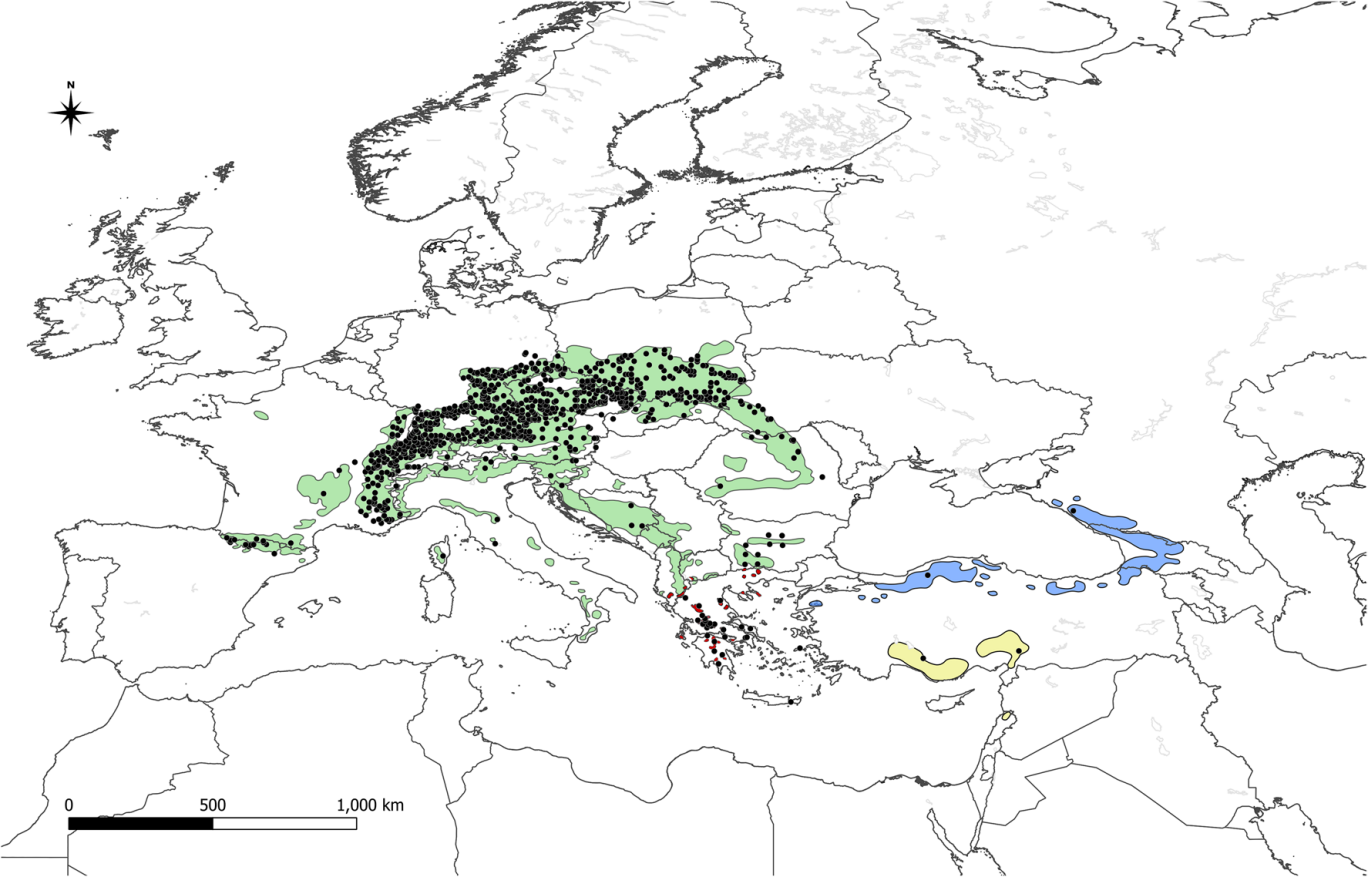
Stands of *Viscum album* subsp. *abietis* used in analyses (black dots) and ranges of its hosts. Green—natural range of *Abies alba;* yellow—natural range of *A. cilicica;* blue—natural range of *A.* *nordmanniana;* red—natural range of *A. cephalonica* and *A. borisii-regis* (Caudullo et al.^70^).

**Figure 3:**
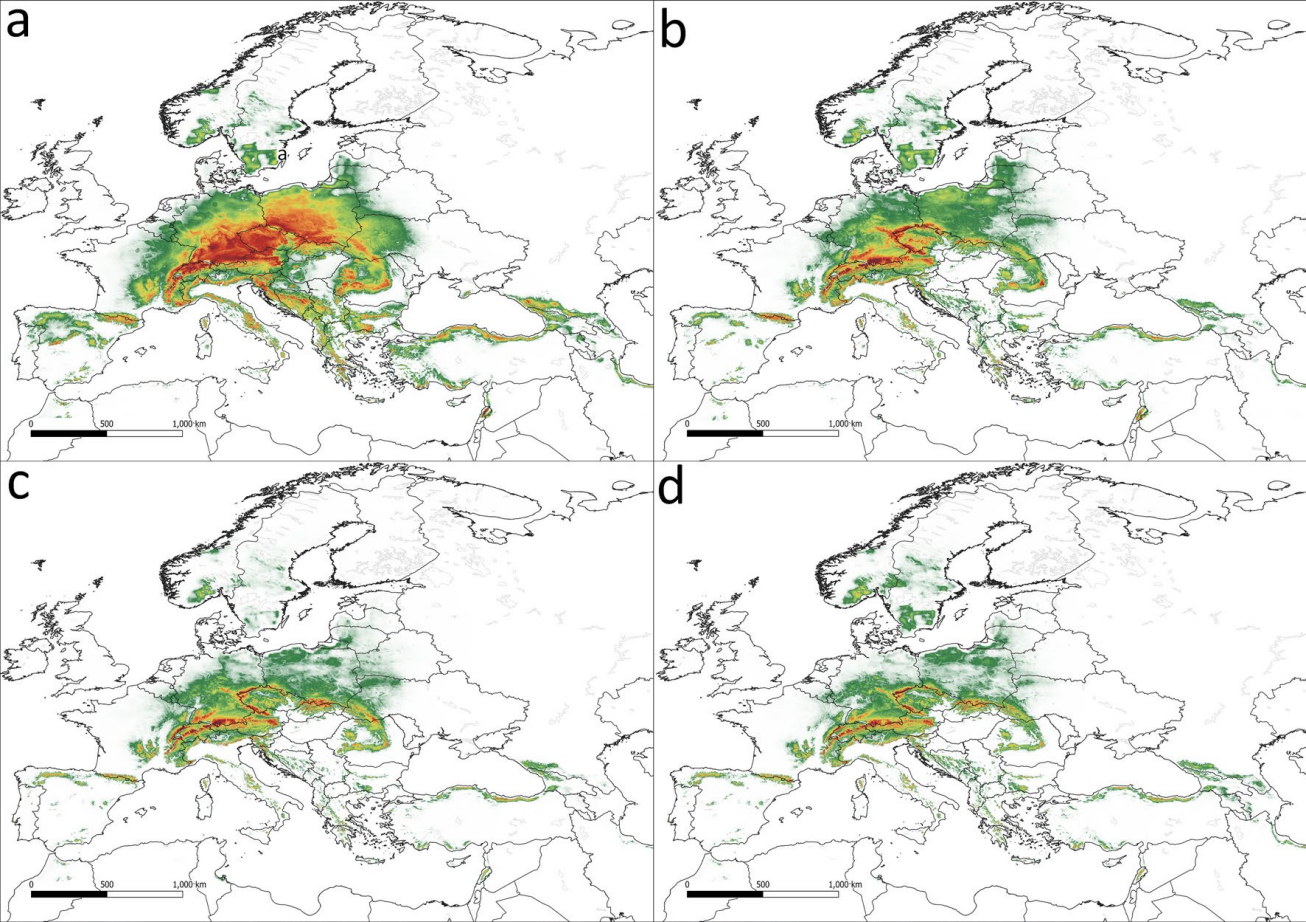
The potential range of *Viscum album* subsp. *abietis*. (**a**) current conditions, (**b**) scenario SSP 1.26, (**c**) scenario SSP 3.7, (**d**) scenario SSP 5.85.

The potential range of *V. album* subsp. *abietis* is concurrent with the range of host species from genus *Abies* (Fig. 2). The potential range covers 1,831,000 km^2^. Under current conditions, the highest suitability has been found in southern Germany, the Czech Republic, Austria, and southern Poland (Fig. 3a). Future climate changes are likely to drastically reduce the potential range of *V. album* subsp. *abietis,* by as much as 61.3% in the SSP 5.85 scenario (Table 2, Fig. 3b–d). Even in the most optimistic scenario (SSP 1.26), the potential range will decrease by 37.5% and high suitability remains only in mountainous areas of Central Europe.

**Table 2:** Suitable geographical area of *V. album* in the tested climate models, divided into four classes according to suitability: low (0.1–0.24), moderate (0.25–0.49), high (0.50–0.74) and very high (≥ 0.75).

Taxa	Scenario	Suitability (area in 10^3^ km^2^)					Change
		Low	Moderate	High	Very high	Sum	
*V. album* subsp. *abietis*	Current	665	559	397	209	1 831	–
	SSP 1.26	647	312	132	54	1 144	− 37.5%
	SSP 3.70	411	210	100	37	757	− 58.7%
	SSP 5.85	433	177	77	22	709	− 61.3%
*V. album* subsp. *austriacum*	Current	1197	852	444	294	2 788	–
	SSP 1.26	1119	836	458	234	2 645	− 5.1%
	SSP 3.70	1243	730	397	128	2 498	− 10.4%
	SSP 5.85	1158	669	314	62	2 203	− 21.0%
*V. album* subsp. *album*	Current	1219	1433	2404	836	5 892	–
	SSP 1.26	1841	1926	2271	994	7 033	+ 19.4%
	SSP 3.70	2482	2609	2340	1070	8 501	+ 44.3%
	SSP 5.85	2076	2060	2206	801	7 143	+ 21.2%

Significant values are in [bold].

**Figure 4:**
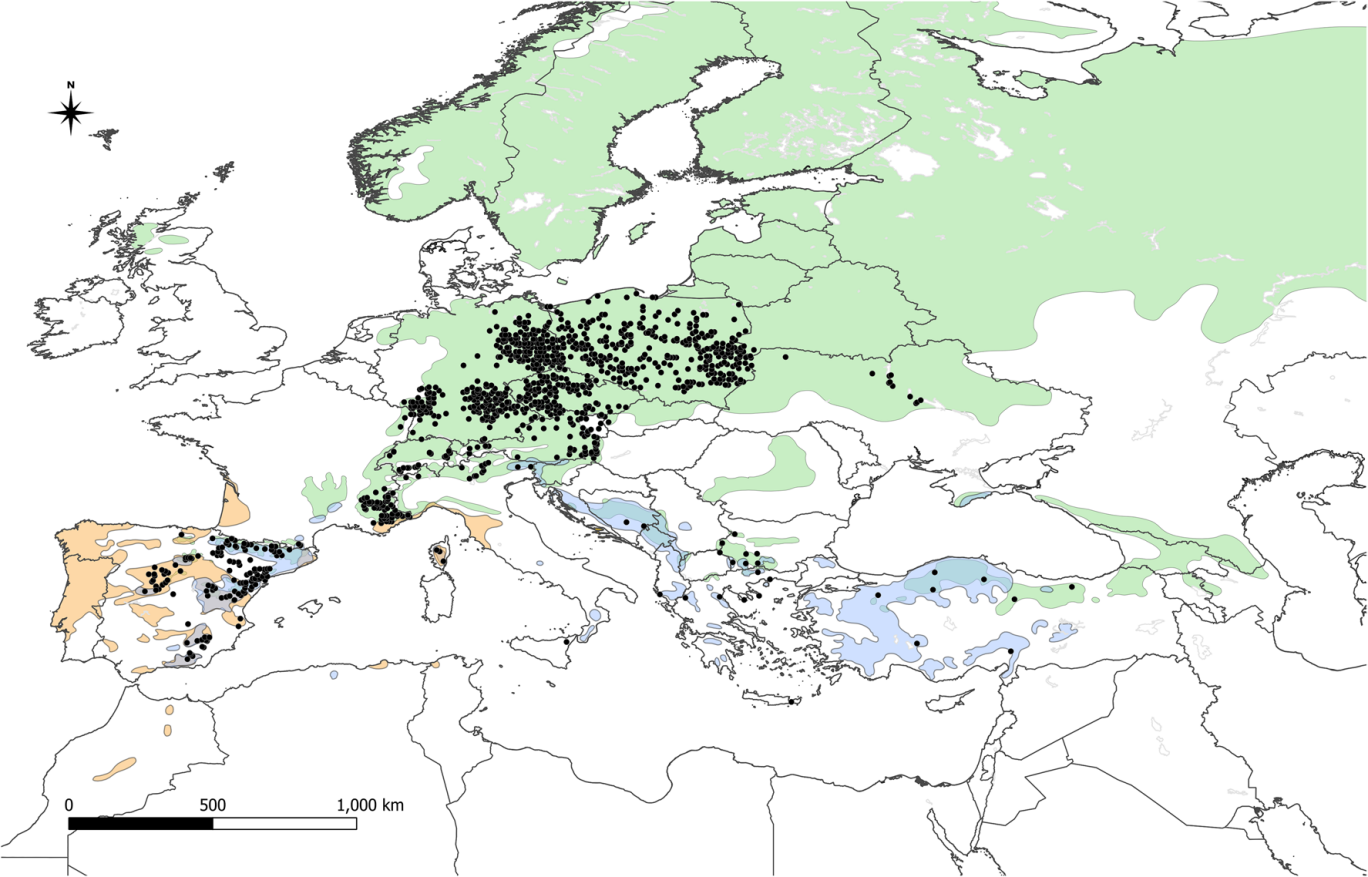
Stands of *Viscum album* subsp. *austriacum* used in analyses (black dots) and ranges of its hosts. Green—natural range of *Pinus sylvestris*; orange—natural range of *P. pinaster;* blue—natural range of *P. nigra* (Caudullo et al.^70^).

**Figure 5:**
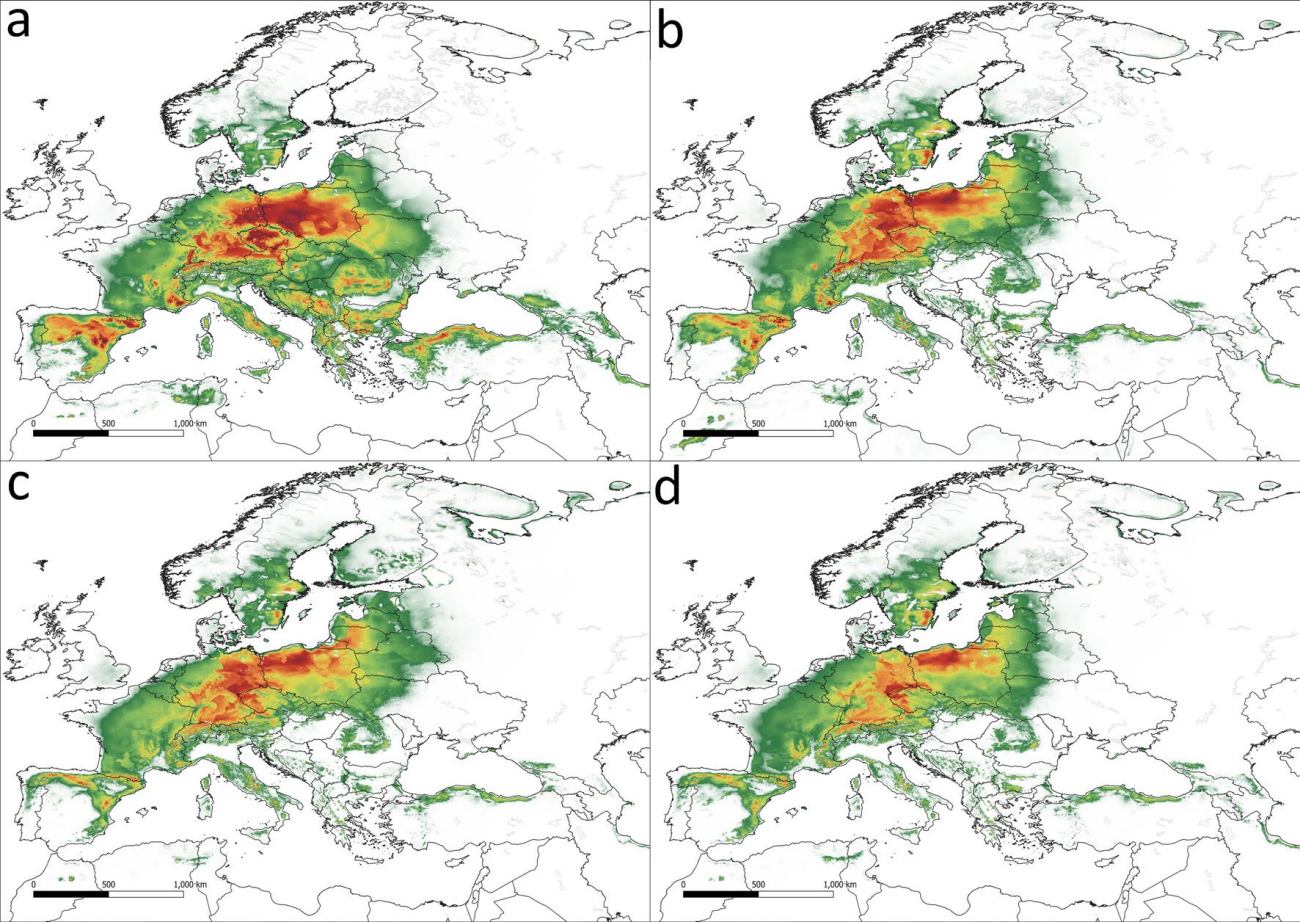
The potential range of *Viscum album* subsp. *austriacum*. (**a**) current conditions, (**b**) scenario SSP 1.26, (**c**) scenario SSP 3.7, (**d**) scenario SSP 5.85.

The second subspecies, *V. album* subsp. *austriacum,* occurs on the trees from genus *Pinus*; however, the range of its main host, *P. sylvestris* is so wide that the host occurrence is probably not a limiting factor in comparison with the case of *V. album* subsp. *abietis* (Fig. 4). The potential range under current conditions covers a large part of the continent (2,788,000 km^2^), but the most suitable area has been detected in Central Europe (Fig. 5a). In future scenarios, this subspecies almost disappears in the Balkan Peninsula, while high suitability will be found in northern Poland, eastern Germany and western part of Czechia (Fig. 5b–d). Moreover, potential suitability in the Iberian Peninsula is very reduced in future scenarios (area with suitability higher than 0.25 will be circa 65% smaller in SSP 5.85 scenario than in the current conditions). Potential range is projected to decrease between 5.1% (SSP 1.26 scenario) and 21% (SSP 5.85).

**Figure 6:**
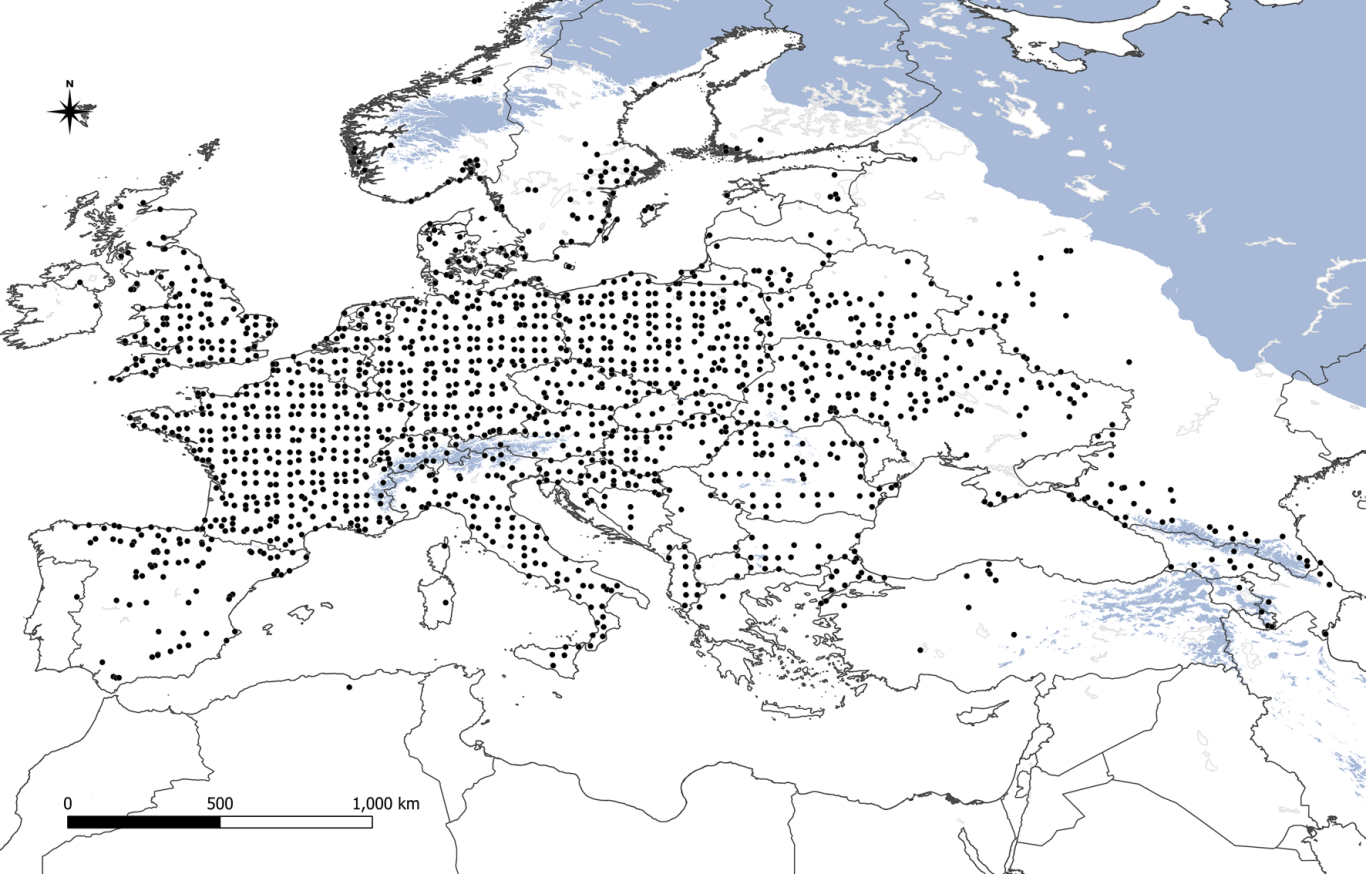
Stands of *Viscum album* subsp. *album* used in analyses (black dots). Blue colour indicates areas with average temperature of January below − 8  °C.

**Figure 7:**
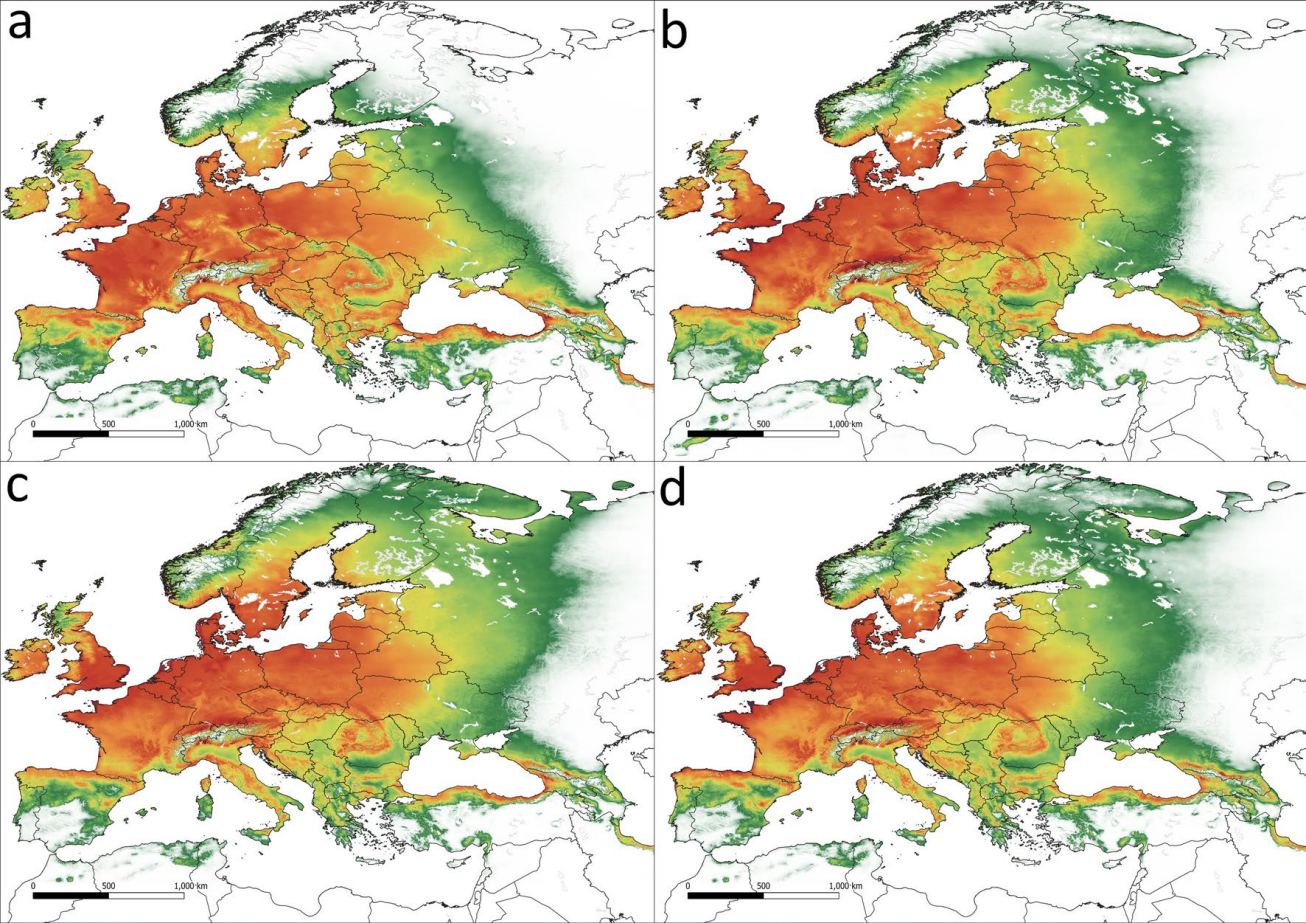
The potential range of *Viscum album* subsp. *album*. (**a**) current conditions, (**b**) scenario SSP 1.26, (**c**) scenario SSP 3.7, (**d**) scenario SSP 5.85.

The most widespread subspecies is *V. album* subsp. *album*, whose potential range covers most of Europe (5,892,000 km^2^, Fig. 6). The current eastern limit of its occurrence is close to the January isotherm of − 8  °C (Fig. 7a). In contrast to the other two subspecies, the potential range of *V. album* subsp. *album* expands under all tested future climate scenarios and shifts northeast, reaching as far as the shores of the White Sea (Fig. 7b–d, Table 2). Totally, the subspecies potential range will increase between 19.4% (scenario SSP 1.26) and 44.3% (scenario SSP 3.70). A decline in suitability is seen mainly in the southeastern part of the potential range, especially in the Balkan Peninsula. The central and western parts of Europe, from Ireland to Poland, the core of the range probably will remain until 2070.

## Discussion

European mistletoe is classified into five subspecies. In the presented work, we focused on the potential range of three major, i.e. widely spread European subspecies. Mistletoe is a parasitic plant, thus its range is strictly connected with the range of its hosts. Despite of this, environmental conditions are probably the most significant factors that influenced the range of the *Viscum album*. In other words, we can say that the mistletoe does not grow in all the areas where its hosts can, or that the presence of the mistletoe is not solely dependent on the presence of its host, because some other factors may limit its occurrence, too. Results of MAXENT modelling suggest that temperature is the key variable, while precipitation is less important, which can be connected with the fact that rain has an indirect impact as the mistletoe takes water from the xylem of the host^25,62^. Previous work has also pointed to temperature being the dominant factor in influencing the range of mistletoe, which occurs in regions with a mean temperature of the coldest month above − 7.7  °C and the average temperature of the warmest month above 15  °C^25,28,30,63^. According to collected data, most stands are located in areas with annual mean temperatures between 5 and 15  °C. From 11 tested bioclimatic variables, one, bio7 (temperature annual range), appeared to be significant for all tested subspecies. Mistletoe prefers areas with a temperature amplitude lower than 30  °C; thus, the suitability for the species is highest in areas of Europe with an oceanic climate, especially in the case of the commonest subspecies, *V. album* subsp. *album*. The significance of other climatic factors varies between tested subspecies, however, these differences can be connected with different, particular requirements of the host tree species, as discussed below.

The potential range of the mistletoe may also be influenced by human activity. An increase in the abundance of mistletoe is associated with planting of parks or parkways with suitable hosts and good light conditions for single or loosely planted trees; it may also be important to note that the urban area has many locations conducive to birds that may spread mistletoe^64,65^. Thus, the conditions for growth and spreading in urban conditions are very good, especially since mistletoe is very resistant to air pollution^66,67^. *Viscum album* subsp. *album* is also more common in the centres of the cities than in the suburban areas^68,69^. Thus, presented models which used bioclimatic factors should be considered with caution, because additional factors can significantly change the real range of analysed species, as selection of the variables is crucial for the model^7^.

The obtained results suggest that the different mistletoe subspecies probably differ in their climatic requirements. The previously described biochemical differences^36^ and the almost complete lack of gene exchange between subspecies^25,35^ makes one wonder whether, despite their high morphological similarity, these three taxa should not be treated as closely related, but separate species. This would be a return to earlier concepts in which mistletoe occurring on pine trees was referred to as *Viscum laxum* Boiss, while mistletoe occurring on trees from the genus *Abies* was referred to as *Viscum abietis* Beck.^25,39^.

### *Viscum album* subsp. *abietis*

Subspecies *Viscum album* subsp. *abietis* occurs widely within the range of its primary host, *Abies alba*^70^. MAXENT results suggest that the potential range of this subspecies is much wider than its actual range, which indicates that the presence of its host probably has a greater, more limiting effect on its geographic range than climatic conditions. This may be the reason why none of the bioclimatic factors was clearly dominant; the most important were temperature annual range and isothermality (a variable that determines the ratio of the diurnal temperature amplitude to the annual temperature amplitude^71^). A significant factor was also the temperature of the coldest month, which is the limiting factor not only for this subspecies but for the whole species at the northeastern edge of its range^25,72^. Limitation of this subspecies to firs may define the altitudinal limit in mountainous areas, especially in the Alps^30^. Interestingly, precipitation of the warmest quarter also had a significant influence on a model (more than 10% of contribution). The tested subspecies occurs in the areas with an average rainfall during the warm season higher than 200 mm. This is probably related to the environmental requirements of the host—mainly *Abies alba*. This climatic factor is significant, and its minimum value is also around 200 mm^3^. In the mountainous regions, aspect also may be significant—tested subspecies is more common on southern and western slopes than on eastern and northern slopes^30^. The results indicate that future climate changes will probably drastically reduce the range of *V. album* subsp. *abietis.* Around the year 2070, the best conditions for this subspecies may be found in the Alps and mountainous areas on the border between the Czech Republic and Germany. Populations from southeast Europe, that occur in dispersed areas often distant from each other, will be endangered^25^. This implies the possibility of significant genetic impoverishment of this subspecies, as populations from Greece and Turkey are represented by different haplotypes than the one that dominates the rest of Europe. Turkish populations (which occurs on *Abies cilicica* (Antoine & Kotschy) Carrière and *Abies bournmuelleriana* Mattf.) may even constitute a distinct taxon^35^. Changes in climatic conditions associated with increasing temperatures and decreasing precipitation will also adversely affect the main host. The potential range of *Abies alba* will be much smaller in the future, especially in the eastern and southern parts of Europe^3,58^. In the 2081, European area with high suitability for the fir may be just over 60% of the current, with the inclusion of potentially suitable areas in the Scandinavia^10^. The presence of mistletoe, which disrupts the water balance of the tree, may further accelerate the withdrawal of fir from areas where there will be a significant increase in value of potential evapotranspiration^19,73^. Weakening of the host trees may cause the expansion of mistletoe, however, the decline of the fir in the long term will pose a threat to the *V. album* subsp*. abietis* itself.

### *Viscum album* subsp. *austriacum*

The potential range of the second subspecies, *Viscum album* subsp. *austriacum,* is much smaller than the range of its most important host, *Pinus sylvestris*. This subspecies probably survived the period of glaciation in the Iberian Peninsula, and from this region recolonized other parts of Europe; however, there is still potential for the spread of this subspecies, because the current estimated range is wider than the actual range^35^. The more reduced range of this subspecies in the north and east, compared to the *V. album* subsp. *album*, may be due to the greater sensitivity to lower temperatures of *V. album* subsp. *austriacum* seeds^74^. In the future, the area suitable for *V. album* subsp. *austriacum* will be reduced. The southernmost, scattered populations on the Balkan Peninsula may disappear; however, in south-western Europe, the decline in suitability will be not as rapid. This result may be explained by the significant influence of continentalism on the subspecies' distribution^37^. At the same time, the potential range will be rather stable in the northern part of Europe, and in some countries (Latvia, Estonia, Sweden) the suitability will be even higher than today, which is likely related to the increase in temperatures in the cold quarter^28^. Our results suggest that *V. album austriacum* may be a serious factor which will negatively affect pine-dominated forests in Central Europe. Already under current conditions, this subspecies is becoming a major problem in pine stands in this region^13,19,75–77^. This problem may be exacerbated by climatic change, weakening host trees. The presence of mistletoe significantly worsens the condition of the tree during the dry summer seasons, because infestation by *Viscum* reduces water and mineral nutrient availability^17,78^. Mistletoe has higher stomatal conductance and transpiration rates than the host, which causes a significant loss of water in the tree and affects its growth^19,79,80^. Sangüesa-Barreda et al.^12^ suggested that mistletoe may be a factor that predisposes pine trees to the effects of drought, causing them to enter a decline process in which other factors, like pests and diseases, may eventually cause tree death. Unfortunately, models of the future potential range of pine predict a significant climate change-induced contraction in all analysed scenarios^3,4^. Unfavourable environmental conditions, combined with the expansion of mistletoe and the occurrence of various pests, may lead to the disappearance of *Pinus sylvestris* in Central Europe, which from the economic point of view, will be accompanied with the need for an overall reconstruction of the forests.

### *Viscum album* subsp. *album*

The most widespread subspecies, *Viscum album* subsp. *album,* is the only tested subspecies, which may significantly widen its range area in the future due to climate changes. Because this taxon occurs on a wide variety of deciduous tree species that represent not only various genera but even families, its range is not essentially limited by the host range^32^. In other words, it may encounter suitable hosts in a great majority of environments throughout Europe. Thus, *V. album* subsp. *album* has a much greater actual range than the other subspecies, covering their occurrences almost completely, except for small areas in Corsica, Spain, Turkey, and the Balkans^25^. The most significant factor which shapes the potential range of the typical subspecies is the minimal temperature of the coldest month (45.5% in the model of current climate). This is consistent with earlier reports in the literature which suggested that temperature is the limiting factor in the north-eastern border of the range of *Viscum* and mistletoe occurs in areas where the mean temperature of January is higher than  − 7.7  °C^25,63,72^. The important impact of the minimum temperature is probably related to the biology of mistletoe, which is an evergreen plant. The temperature of the coolest (as well as the warmest) month is related with respiration equivalent (heat sum that is necessary for the respiration of evergreen plants^63,81^). This factor is calculated by weighting the hourly temperatures (according to relative respiration at a given temperature level) and summing them over the period considered^82^. Future climate changes, which are expected to cause the temperature rise, may trigger the expansion of mistletoe in Scandinavia and Northern Europe. Mistletoe was more common in Scandinavia during the warm period of the Holocene, and in few sites in Sweden, it has survived to the present day^29,83^. An analysis that used temperature as the only factor influencing the species range showed that rising temperatures in both summer and winter seasons can cause mistletoe to thrive again in Scandinavia, while disappearing in the Mediterranean region^28^. A similar effect was pictured in the presented MAXENT model which used a set of bioclimatic variables. The fact that this subspecies is likely to expand in the future, may be facilitated by several additional factors, not directly related to climatic conditions. Firstly, the large number of host species practically does not limit the potential range because the hemi-parasite may encounter a suitable host plant almost everywhere. Secondly, bird species that feed on mistletoe and cause it to spread (like *Turdus viscivorus* L., *Turdus pilaris* L.*, Bombycilla garrulus* L.) regularly migrate through Europe^23,25,50^. Dispersal of mistletoe by birds is crucial for the spread and survival of this species^23,76^. Observational studies showed that *Turdus viscivorus* is the most effective seed disperser. It means that the species provides the highest probability that the seeds will be carried by a bird to the "safe place". Opportunistic species from genus *Parus* were the most efficient seed dispersers (highest number of seeds dispersed relative to seeds handled^23^). Blackcap *Sylvia atricapilla* L. can also be considered a relevant vector^23^. Although seed dispersal by consumption occurs over short distances of up to 20 km, the eventual sticking of the seed to the bird's feathers, due to the sticky coat, may allow dispersal over long distances^35^. Because mistletoe seeds have a high germination rate, when brought by birds to a new area they can easily become the beginning of a new population^84^. The typical subspecies of *Viscum album* is characterised not only by a large number of potential hosts and relatively easy seed dispersal, but also by high genetic variability, especially in Eastern Europe^35^. Rich gene pool is crucial for the evolutionary potential and adaptation to changing environmental conditions. Some impediments to migration of the *V. album* subsp. *album* to north and east Europe may be the fact that coniferous forests dominate there, which limits the potential host pool for typical subspecies of *V. album*.

## Conclusions

Mistletoe is an important species for several reasons; it can be seen both as a threat to forest trees and as a valuable medicinal plant used in pharmacy. The results obtained in the presented work allow us to estimate the dynamics of mistletoe range changes and plan an appropriate strategy related to forest management. The potential range of mistletoe will shift in a north-eastern direction, while in the mountainous areas of Europe—to higher altitudes. The presence of mistletoe may negatively affect forest communities in Central and Eastern Europe, accelerating the trees' dieback (of particular importance may be the subspecies *V. album* subsp. *austriacum*, which poses a threat to pine-dominated forest complexes). On the other hand, mistletoe populations from South Europe, which are genetically distinct and could be a source of pharmaceutical raw materials, may be lost. The estimated range changes are based on climatic data, but it should be remembered that other factors, such as seed dispersal by birds, also affect the spread of mistletoe; however, data on mistletoe seed dispersal and germination are still quite limited, so there is a need for additional research.

## Data Availability

Data analyzed during the current study are available upon reasonable request from the corresponding author.
